# Treatment Expectations, Convenience, and Satisfaction with Anticoagulant Treatment: Perceptions of Patients in South-East Queensland, Australia

**DOI:** 10.3390/jcm8060863

**Published:** 2019-06-17

**Authors:** Jonathan Gospos, Nijole Bernaitis

**Affiliations:** School of Pharmacy & Pharmacology and Quality Use of Medicines Network, Griffith University, 4222 Queensland, Australia; jonathan.gospos@griffithuni.edu.au

**Keywords:** oral anticoagulant, warfarin, non-vitamin K oral anticoagulant, patient satisfaction

## Abstract

Background: Warfarin has long been the only oral anticoagulant (OAC) available, but options now include non-vitamin K antagonists. Prescribing an OAC may be influenced by patient factors and preferences influenced by dosing, monitoring, and adverse effects, which may ultimately impact patient satisfaction and convenience. The aim of this study was to explore the perception of OAC treatment by Australian patients in terms of treatment expectations, convenience, and satisfaction. Methods: The Perception of Anticoagulant Treatment Questionnaire was distributed to patients dispensed OAC medication from three pharmacies in South-East Queensland. Responses to questions using a five-point Likert scale were collated and mean results utilised to assess expectations, convenience, and satisfaction, including an analysis across demographic groups. Results: A total of 56 (26.8%) surveys were returned, with the majority of respondent’s male (58.2%). Highest mean scores for treatment expectation were for an OAC that was easy to take (4.85 ± 0.79) and that could be taken care of by the respondents themselves (4.11 ± 1.14). The mean overall score for convenience was 68.90 ± 11.44% and for satisfaction 69.43 ± 16.58%. Significantly higher mean convenience scores were found in females and patients with atrial fibrillation. Conclusions: Patients’ highest expectations were for an OAC that would be easy to take, and overall satisfaction and convenience was around 69%. Factors including demographics can influence perceptions of therapy, and addressing individual preferences for OAC therapy may increase ratings of satisfaction and convenience.

## 1. Introduction

Oral anticoagulants (OACs) are prescribed for a number of indications, including atrial fibrillation (AF), venous thromboembolism (VTE), and myocardial infarction (MI) [1]. Available anticoagulant therapy now includes non-vitamin K antagonists (NOACs), such as dabigatran, rivaroxaban, and apixaban, in addition to vitamin K antagonists (VKA) such as warfarin. Selection of the most suitable OAC for a patient involves the assessment of co-morbidities, concurrent medication, and the risk of complications including bleeds [2]. Additional patient factors such as gender and age also require consideration in assessing the risk-benefit of OAC therapy [3]. However, whilst treatment decisions regarding OACs need to consider clinically relevant patient characteristics, consideration must also be given to patient preferences [4]. Andrade et al. [5] found patients and physicians differ in their ranking of ideal characteristics of OAC medications. Similarly, Palacio et al. [6] found values considered important differed from patients to physicians but regardless of this, 85% of patients wanted to actively participate in decisions regarding anticoagulation.

Patient preferences for OAC are influenced by factors including bleed risk, adverse effects, dosing frequency, dietary restrictions, and administration in relation to food [7]. Further to this, Böttger et al. [8] found patients preferred a once daily intake of OAC and no need for monitoring or dose adjustment. Convenience has been reported as a priority for patients [9] and a factor that may influence satisfaction with OAC treatment [10]. Benzimra et al. [11] found patients reported greater satisfaction and convenience when taking NOACs compared to VKAs to treat AF. However, an Australian study of patients with AF by Obamiro et al. [12] found no significant differences in treatment convenience or satisfaction between patients taking NOACs or warfarin. Similar to this, another Australian study by Bajorek et al. [13] found patients with AF were satisfied with their current treatment, whether warfarin or NOAC, and had a preference to remain on existing therapy rather than change agents. Gebler-Hughes et al. [14] also determined that almost 80% of Australian patients were satisfied with warfarin therapy, but this was in 2013 when NOACs were only being first introduced into the market. Thus, the limited number of studies from Australia have focussed on OAC knowledge in patients with AF [12] and assessed patients’ preferences for NOACs compared to warfarin [13,14]. Therefore, the overall aim of this study was to further explore patient perceptions of OAC therapy prescribed for any indication, in terms of patient treatment expectations, convenience, and satisfaction for patients from South-East Queensland, Australia.

## 2. Experimental Section

### 2.1. Survey

Griffith University ethics was obtained (GU 2018/905). The validated Perception of Anticoagulant Treatment Questionnaire (PACT-Q) was utilised to evaluate expectation, convenience, and satisfaction with anticoagulant therapy. The PACT-Q1 questionnaire assesses treatment expectation via seven questions, with participants selecting responses from a five-point Likert scale (1 = not at all, 5 = extremely). The PACT-Q2 questionnaire consists of thirteen questions relating to treatment convenience and seven relating to treatment satisfaction. Responses from each question on a five-point Likert scale are utilised to calculate a global dimension score (maximum 100) for both convenience and satisfaction.

### 2.2. Recruitment

Participants were identified via dispense records from three community pharmacies on the Gold Coast, namely, Surfers Paradise 24hr Chempro Chemist, Ashmore Plaza Chempro Chemist, and Bronberg Plaza Chempro Chemist. People who had been dispensed oral anticoagulants from these pharmacies were supplied the questionnaire, a demographic sheet, and an accompanying information sheet. Informed consent was implied by return of the completed survey via supplied reply-paid envelopes. To maintain anonymity, address labels were generated in the pharmacy, attached to pre-prepared packs containing no identifying information, and immediately posted. PACT-Q1 and PACT-Q2 were distributed by post to participants together with a patient demographic sheet requesting information such as gender, age, highest level of education, occupation, diagnosis for anticoagulant medication, and currently prescribed anticoagulant.

Statistics from the Pharmaceutical Benefits Scheme indicate over one million prescriptions in Queensland for anticoagulants from an overall population of over five million [15]. A power calculation with an 80% confidence interval and a 5% error risk identified that a sample size of 164 surveys was required for results to be generalisable to the entire Queensland population taking oral anticoagulants.

### 2.3. Statistical Analyses

Categorical variables were represented as number and percentage and continuous variables represented as mean and standard deviation. A one sample t-test was performed on responses to each question in PACT-Q1 and global dimension scores to determine overall participant mean and standard deviation for these values. Demographic data was used to categorise patients and compare responses between groups via a two-sample independent t-test. Categories included gender of male versus female, age of ≥75 years versus ≤74 years, highest education high school/primary school versus university/college/other institute, and diagnosis of AF versus other diagnosis. Statistical analyses were conducted using Statistical Package for the Social Sciences (SPSS) Version 25, with a *p*-value of <0.05 considered statistically significant.

## 3. Results

A total of 209 surveys were posted to potential participants, with 10 of these undeliverable by the postal service. A total of 56 surveys were returned completed (26.8% response rate), with the demographic section not completed for one survey. The majority of respondents were male (58.2%), less than 75 years of age (56.4%), and retired (85.5%) (Table 1). The main indication for oral anticoagulant therapy was AF (56.4%), with rivaroxaban most commonly used (45.5%) followed by apixaban (27.3%) and warfarin (16.4%).

Responses regarding treatment expectation resulted in the highest mean scores for having an anticoagulant that is easy to take (4.85 ± 0.79) and that can be taken care of by respondents themselves (4.11 ± 1.14) (Table 2). The lowest mean score was in response to concerns about making mistakes when taking anticoagulant treatment (1.86 ± 1.31), but there were significantly higher mean scores for males compared to females (2.26 ± 1.53 vs. 1.38 ± 0.77, *p* = 0.012) and for respondents with other diagnosis compared to a diagnosis of AF (2.29 ± 1.52 vs. 1.55 ± 1.06, *p* = 0.037). A significant difference in responses was found in relation to concern about how much payment was required for anticoagulant treatment by participants whose highest education level was primary/high school, compared to university/college/institute (3.10 ± 1.58 vs. 2.16 ± 1.38, *p* = 0.024) graduates. No other significant differences were found in demographic comparison of treatment expectation questions.

The PACT-Q global dimension score was 68.90 ± 11.44% for convenience and 69.43 ± 16.58% for satisfaction (Table 3). Significantly higher mean convenience scores were found in females compared to males (72.54 ± 7.84% vs. 65.97 ± 13.31%, *p* = 0.037) and respondents with a primary diagnosis of AF compared to other diagnosis (72.26 ± 10.71% vs. 64.42 ± 11.49%, *p* = 0.012). No significant differences were found in global satisfaction scores from demographic comparisons.

## 4. Discussion

Oral anticoagulant treatment options have increased since the introduction of the NOACs, resulting in higher prescribing rates of OACs in many countries, including Australia [16]. Numerous patient factors must be considered when prescribing anticoagulation [4], but the characteristics of the chosen OAC can potentially contribute to patient satisfaction and convenience with OAC therapy [8,9,10,11]. The aim of this study was to explore treatment expectations, convenience, and satisfaction with OAC in patients from South-East Queensland, Australia. This study found patients convenience and satisfaction scores were around 69%, and their greatest expectation was of an OAC that was easy to take and could be managed independently.

In this study, the highest expectation scores were for an OAC that was easy to take and that could be taken independently. This was in accordance with Obamiro et al. [12], Cajfinger et al. [17], and Larochelle et al. [18], who utilised the PACT-Q1 survey and reported the highest mean expectation scores in having an OAC that was easy to take. In addition, these three studies [12,17,18] reported the lowest expectation score related to concerns about making mistakes, which is consistent with our study. Comparable to these findings, Smet et al. [19] found the highest expectation scores with ease of use and independence, but the lowest with mistakes and costs. Cajfinger et al. [17] also reported low expectation scores regarding cost, whereas our participants were only moderately concerned regarding cost. In Australia, medications are subsidised by the Australian Government under the Pharmaceutical Benefits Scheme, and currently four OAC (warfarin, dabigatran, rivaroxaban, and apixaban) are available and subsidised in Australia [15]. International health systems vary with regard to access and costs for patients, which can impact public views [20]. This may explain differences found in regard to costs across studies from different countries. In this study, cost was of moderate importance but the respondents with lower education levels were significantly more concerned about costs than university or college graduates. This may be attributed to university or college graduates having higher socioeconomic status [21] or higher paying jobs [22], and thus being less concerned about costs. In this study, another demographic factor that significantly influenced treatment expectation was gender, with males more concerned about making mistakes with OAC treatment. Gender can influence the Health Belief Model [23] but DiMatteo [24] reviewed fifty years of research and found overall there was virtually no correlation with gender and adherence. However, Courtenay et al. [25] found studies consistently showed being female was the strongest predictor for health-promoting behaviour. The likelihood that females may by more likely to adhere to treatment by consistently taking prescribed therapy may partly explain the lower concern expressed by females about missing doses of OAC.

In this study, both the global convenience and satisfaction score was around 69%. Satisfaction scores from previous studies have been similar to this, with Obamiro et al. [12] reporting 68.6% and Smet et al. [19] and Cajfinger et al. [17] reporting slightly lower scores of 62.3% and 62.9%, respectively. Similar to this, Benzimra et al. [11] reported scores ranging from 62% with warfarin to 74% with NOACs. Other studies also reported differences in satisfaction between VKA and NOACs, with Keita et al. [26] reporting 88% satisfaction with NOAC therapy and 81.5% with VKA, whereas Goette et al. [27] reported satisfaction scores of 65.8% for patients taking edoxaban and 70.6% for those on enoxaparin/warfarin. In contrast, Okumura et al. [28] found no difference in global satisfaction scores between NOAC and warfarin groups of 65% and 66%, respectively, but suggested that NOAC users had greater satisfaction relating to burden of treatment. In contrast, Bajorek et al. [13] found the burden of warfarin monitoring may not impact patient satisfaction and found warfarin-treated patients often perceived the lack of monitoring with NOACs to be a deterrent to use. Further to this, Ikeda et al. [29] found approximately 50% of patients offered to change from warfarin to an NOAC elected not to for reasons including long-term positive experiences with warfarin. Similarly, Wiley et al. [30] determined that patients were more willing to switch to an NOAC if satisfaction with warfarin was low. DeCaterina et al. [31] found patients switched to NOAC were more often dissatisfied with the OAC treatment, and studies [32,33,34] have also reported between 5 and 13% of patients converting from warfarin to NOAC actually returned to warfarin therapy for reasons including intolerance or bleeds. Further to this, MacLean et al. [35] found patient preferences in therapy may depend on their prior experience with the treatment. Weernik et al. [9] found that adverse effects impact perceptions of OAC treatment, whilst Hellfritzsch et al. [36] found the greatest issues surrounding OAC therapy were adverse effects and inconvenience.

The mean global convenience score in this study was 68.9%. Higher convenience scores have been reported by Cajfinger et al. [17] with 79.7%, Smet et al. [19] with 86.7%, and Obamiro et al. [12] with 88.4%. Interestingly, Obamiro et al. [12] reported the highest convenience score but also had a higher proportion of females (68%) than our study, and we found significantly higher convenience scores in females. The different demographic profiles of these two studies could explain some of the difference in overall convenience scores. In our study, the majority (80.1%) of participants were NOAC users. Higher convenience scores with NOACs have been reported by both Benzimra et al. [11] with 96% with NOAC compared to 87% with VKA, and Goette et al. [27] of 86.4% with edoxaban compared to 82.7% with enoxaparin/warfarin. Further to this, Choi et al. [10] reported greater convenience in dabigatran compared to warfarin users. Rivaroxaban and dabigatran may be dosed once or twice daily depending on indication, whereas dosing is fixed for apixaban as twice daily and edoxaban once daily [37]. Warfarin is dosed only once daily but requires frequent monitoring [38], which affects the convenience of dosing. Gebler-Hughes et al. [14] reported only around 7% of patients disliked the regular monitoring with warfarin, whereas Benzimra et al. [11] reported 50% of patients would switch to an NOAC to avoid blood tests. However, Alegret et al. [39] observed concern regarding distress and daily hassles improved in a VKA group over time. Gadisseur et al. [40] reported patient distress associated with frequent warfarin appointments could be reduced by patient self-monitoring with Grove et al. [41], suggesting self-managed warfarin could be an effective alternative to NOAC treatment. Arnsten et al. [42] correlated inadequate warfarin monitoring with younger age, while both Luger et al. [43] and Alamneh et al. [44] found younger age was a strong factor for prescribing NOAC instead of warfarin. Further to this, Gebler-Hughes et al. [14] found increasing age correlated with greater satisfaction with oral anticoagulant therapy. In this study, we found age was not a factor for either convenience or satisfaction but patients with AF rated convenience of therapy significantly higher than patients with other diagnoses. Both MacLean et al. [35] and Alegret et al. [39] reported decreased aversion to warfarin treatment over time, whilst Gebler-Hughes et al. [14] suggested chronic conditions can impact the need for medical visits, thereby reducing the apparent burden due to warfarin monitoring. Furthermore, a review by Jin et al. [45] suggested longer duration of disease may improve patient compliance due to acceptance of treatment required for chronic disease. Therefore, the long-term OAC therapy required for AF compared to shorter duration of treatment for diagnosis such as DVT may partly explain the higher satisfaction scores in patients with AF. However, a limitation of this study was the small sample size that influenced the ability to adequately analyse confounding factors. With only 55 respondents, the study power was reduced, and hence results are not generalisable to the entire Queensland population. In addition, the small number of patients taking warfarin limited the ability to compare preferences between patients taking warfarin and NOACs. Future studies should consider alternate recruitment methods such as survey completion in the pharmacy or online surveys to obtain higher response rates and participants from more geographically diverse populations. It would also be beneficial to capture further information regarding the OAC therapy, including length of treatment, past therapy, and current dosing, as this may influence perceptions of satisfaction and convenience with therapy.

## 5. Conclusions

In conclusion, this study found patients rated their satisfaction and convenience with OAC at around 69%. The greatest expectation of patients in this study was for an OAC that would be easy to take and could be managed independently. Expectations and perceptions of convenience were influenced by gender and diagnosis of AF. Discussing specific concerns with patients may assist in selecting an OAC that aligns with individual patient preferences and subsequently improves these perceptions.

## Figures and Tables

**Table 1:** Demographic profile of participants (*n* = 55).

Patient Characteristic	Number (Percentage)
**Gender**	
Female	23 (41.8)
Male	32 (58.2)
**Age**	
≤74 years	31 (56.4)
≥75 years	24 (43.6)
**Highest Level of Education**	
Primary School	3 (5.4)
High School	27 (49.1)
College/Institute	16 (29.1)
University	9 (16.4)
**Occupation**	
Retired	47 (85.5)
Employed	7 (12.7)
Disabled	1 (1.8)
**Diagnosis**	
Atrial fibrillation	31 (56.4)
Deep vein thrombosis	4 (7.3)
Myocardial infarction	2 (3.6)
Stroke	2 (3.6)
Pulmonary embolism	1 (1.8)
Other/combination of diagnoses	15 (27.3)
**Medication**	
Rivaroxaban	25 (45.5)
Apixaban	15 (27.3)
Warfarin	9 (16.4)
Dabigatran	4 (7.3)
Other	2 (3.5)

**Table 2:** Responses to treatment expectation questions from PACT-Q1 survey reported as mean (standard deviation) from responses to a five-point likert scale with 1 = not at all, 2 = a little, 3 = moderately, 4 = a lot, and 5 = extremely.

PACT-Q1 Treatment Expectations Questions	Mean (SD)
A1—How confident are you that your anticoagulant treatment will prevent blood clots? (*n* = 55)	4.04 (0.79)
A2—Do you expect that your anticoagulant treatment will relieve some of the symptoms you experience? (*n* = 52)	2.94 (1.28)
A3—Do you expect that your anticoagulant treatment will cause side effects such as minor bruises or bleeding? (*n* = 54)	3.04 (1.21)
A4—How important is it for you to have an anticoagulant treatment that is easy to take? (*n* = 55)	4.85 (0.79)
A5—How concerned are you about making mistakes when taking your anticoagulant treatment? (*n* = 56)	1.86 (1.31)
A6—How important is it for you to take care of your anticoagulant treatment by yourself? (*n* = 56)	4.11 (1.14)
A7—How concerned are you about how much you may have to pay for your anticoagulant treatment? (*n* = 56)	2.64 (1.55)

**Table 3:** Global dimension scores for treatment convenience and satisfaction from PACT-Q2 survey reported as mean (standard deviation) and statistical significance shown between variable categories.

Global Dimension Score	Convenience		Satisfaction	
	68.90 (11.44)		69.43 (16.58)	
**Age**				
≤74 years	68.42 (10.02)		69.38 (13.42)	
≥75 years	68.95 (13.19)	*p* = 0.910	69.89 (19.66)	*p* = 0.917
**Gender**				
Female	72.54 (7.84)		70.58 (13.73)	
Male	65.97 (13.31)	*p* = 0.037	68.01 (18.87)	*p* = 0.576
**Diagnosis**				
Atrial fibrillation	72.26 (10.71)		67.97 (18.58)	
Other	64.42 (11.49)	*p* = 0.012	70.63 (14.24)	*p* = 0.563
**Highest Education**				
High School/primary school	67.13 (11.56)		70.19 (16.33)	
University/college/institution	70.89 (11.64)	*p* = 0.236	67.85 (17.45)	*p* = 0.610
**Medication**				
Rivaroxaban	70.48 (12.44)		67.85 (16.79)	
Other	67.47 (10.94)	*p* = 0.344	70.19 (16.88)	*p* = 0.610
